# Supplementary material for: Rapid, B1‐insensitive, dual‐band quasi‐adiabatic saturation transfer with optimal control for complete quantification of myocardial ATP flux
Source: Magn Reson Med. 2021 Feb 3;85(6):2978–91. doi: 10.1002/mrm.28647 (PMC7986077; doi:10.1002/mrm.28647)
Supplement: Supplementary file 1 — TABLE S1 Summary uncertainty values in derived concentrations TABLE S2 Summary of parameters (Heart 1) TABLE S3 Raw metabolite MRS peak amplitudes and CRLBs, and determined metabolite concentration and Δ Concentration. (Heart 1) TABLE S4 Summary of parameters (Heart 2) TABLE S5 Raw metabolite MRS peak amplitudes and CRLBs, and determined metabolite concentration and ΔConcentration. (Heart 2) TABLE S6 Summary of parameters (Heart 3) TABLE S7 Raw metabolite MRS peak amplitudes and CRLBs, and determined metabolite concentration and ΔConcentration. (Heart 3) TABLE S8 Summary of parameters (Heart 4) TABLE S9 Raw metabolite MRS peak amplitudes and CRLBs, and determined metabolite concentration and ΔConcentration. (Heart 4) TABLE S10 Summary of parameters (Heart 5) TABLE S11 Raw metabolite MRS peak amplitudes and CRLBs, and determined metabolite concentration and ΔConcentration. (Heart 5) [file MRM-85-2978-s001.pdf]

Rapid, B1-insensitive, dual-band quasi-adiabatic saturation transfer with optimal control for complete quantification of myocardial ATP flux

Online Supporting Information

1 Error propagation

1.1 Introduction

It is a well-known fact that phosphorus spectroscopy is unfortunately a fundamentally low SNR technique, owing to the Boltzmann distribution for the ^{31}P nucleus, its lower gyromagnetic ratio than protons, and the lower natural biological concentration than, say, water protons. Furthermore, as outlined in the main text, the experiments performed on perfused hearts are subject to additional sources of biological variability: both individually, each heart will be different, and mechanistically, as their function throughout the perfusion experiment slowly deteriorates at a different rate.

As the work presented here encompasses both forward and reverse saturation-transfer experiments, which necessarily involve scanning a perfused heart for a comparatively long period of time in order to obtain a timecourse of spectral curves under varying saturation durations, it may well be the case that the physiological state of flux balance is slightly different between the forward and reverse experiments. An alternative hypothesis, explored here, is that the nonlinear propagation of uncertainty can combine unfavourably to make the uncertainty on the resulting point estimate potentially larger than it otherwise would be.

1.2 Cramér-Rao Lower Bounds and functions of a random variable

A result from information theory is that the *minimum* bound on an estimator in a statistical setting can be computed from the Hessian of the Fisher information matrix determined in that setting, known as the Cramér-Rao Lower Bound (CRLB). For the case of spectral fitting, particularly where estimates are noisy such as in phosphorus spectroscopy, it is appropriate to take an individual result of a spectral quantification algorithm as a point estimate, x, and the CRLB on x as a measure of its uncertainty, σ_x^2. Typically x is a peak amplitude of interest, though it may be, for example, spectral location.

Under these circumstances, it is appropriate to consider x as a random variable; $x \sim N(\mu_x, \sigma_x^2)$, with the point estimate of x, μ_x that being returned from

a spectral fitting algorithm, and width its CRLB. The main point of this supplement is to briefly highlight that many nonlinear transformations are undertaken between spectral amplitudes, derived concentrations, and the ultimate recorded value of a rate constant.

Firstly, note that taking functions of two random variables, say, x and y and $f(x, y)$, produces a value that is itself randomly distributed. One can appeal to Taylor series expansions, and say that if μ_x is large in comparison to σ_x, and similarly for y, then it will be the case that, if higher order terms are truncated,

$$f = f(x, y); \qquad x \sim N(\mu_x, \sigma_x^2); \quad y \sim N(\mu_y, \sigma_y^2) \tag{1}$$

$$\sigma_f^2 \approx \left|\frac{\partial f}{\partial x}\right|^2 \sigma_x^2 + \left|\frac{\partial f}{\partial y}\right|^2 \sigma_y^2 + 2\frac{\partial f}{\partial x}\frac{\partial f}{\partial y}\sigma_{xy}, \tag{2}$$

where σ_{xy} represents the covariance between x and y, related to a correlation coefficient ρ by $\sigma_{xy} = \sigma_x \sigma_y \rho$. The determination of this is ultimately an experimental question: it can be determined from the Fisher information matrix of the nonlinear spectral fit.

This expression, Eq. (2), is straightforward both analytically and computationally, but it is a simplified approximation. In particular, the truncation of the series expression can produce errors, and the assumptions of smoothness and differentiability that it predicts are not necessarily always true. *If* the uncertainty in x and y are small, it may well be appropriate to consider the ratio a Gaussian PDF with mean $f(x, y)$ and variance σ_{xy}^2, depending upon the choice of f. However, this relies upon the denominator being far from zero. A classic 'pathological' example would be the Cauchy distribution, i.e.

$$f(x, y) = \frac{x \sim N(0, 1)}{y \sim N(0, 1)} \tag{3}$$

which is a distribution with no well-defined mean and variance as the relevant generating moments are described by integrals that do not exist. The analytical form for the ratio distribution can be derived. It is a moderately complex expression: if we define the function of the ratio, $w = x/y$ where x and y have a correlation coefficient ρ, the ratio distribution is given by the expression:

$$\begin{aligned} f(w) = {} & \frac{b(w)d(w)}{\sqrt{2\pi}\sigma_x\sigma_y a^3(w)} \left(\Phi\left(\frac{b(w)}{\sqrt{1-\rho^2}a(w)}\right) - \Phi\left(-\frac{b(w)}{\sqrt{1-\rho^2}a(w)}\right)\right) \\ & + \frac{\sqrt{1-\rho^2}}{\pi\sigma_x\sigma_y a^2(w)} \exp\left(-\frac{c}{2(1-\rho^2)}\right), \end{aligned} \tag{4}$$

where

$$a(w) = \left(\frac{w^2}{\sigma_x^2} - \frac{2\rho w}{\sigma_x \sigma_y} + \frac{1}{\sigma_y^2}\right)^{\frac{1}{2}} \tag{5}$$

$$b(w) = \frac{\mu_x w}{\sigma_x^2} - \frac{\rho(\mu_x + \mu_y w)}{\sigma_x \sigma_y} + \frac{\mu_y}{\sigma_y^2} \tag{6}$$

$$c = \frac{\mu_x^2}{\sigma_x^2} + \frac{2\rho \mu_x \mu_y}{\sigma_x \sigma_y} + \frac{\mu_y^2}{\sigma_y^2} \tag{7}$$

$$d(w) = \exp\left(\frac{b^2(w) - c(w)a^2(w)}{2(1-\rho^2)a^2(w)}\right); \tag{8}$$

and

$$\begin{aligned} \Phi(z) &= \frac{1}{\sqrt{2\pi}} \int_{-\infty}^{z} e^{-\frac{1}{2}u^2}\,\mathrm{d}u \\ &= \frac{1}{2}\left(1 + \mathrm{Erf}\left(\frac{z}{\sqrt{2}}\right)\right), \end{aligned} \tag{9}$$

where Erf(z) denotes the Gaussian error function, $\mathrm{Erf}(z) = \frac{2}{\sqrt{\pi}} \int_0^z e^{-t^2}\,\mathrm{d}t$, and $w = \frac{x \sim \mathcal{N}(\mu_x, \sigma_x)}{y \sim \mathcal{N}(\mu_y, \sigma_y)}$.

This matters in the context of phosphorus spectroscopy, because quite often we compute the ratios of peak amplitudes. In the case that quantities measured are far from zero, the Gaussian approximation is approximately correct, and quite useful: see elsewhere for a discussion of how properly incorporating such information about the expected distribution of the PCr/ATP ratio can improve statistical power when comparing groups of measured ratios.[1]

1.3 Quantification

In this work, the developed saturation pulse was applied in forward and reverse schemes. Absolute quantification was performed on the basis of three separate measurements of the PPA balloon with differing volumes of PPA. This is because it is necessary to inflate the balloon appropriately to fill the LV cavum and this varies from heart to heart. At the end of the experiment, 100 µl of PPA was added, a hard pulse/acquire scan obtained, and then the process repeated once more. This then yields three PPA scouts with differing volumes (but known concentrations) of PPA, from which linear regression provides an estimate of the gradient of signal of PPA to µl. The CRLB on the PPA quantification is neglected, as it is a large peak. It is possible to perform robust linear regression taking into account uncertainty on the y-axis (i.e. incorporating this information), through means such as Deming regression, but that was not performed here. This regression yields a coefficient b, of units au/µl, that converts from PPA to arbitrary units. There is a statistical regression error on this coefficient; its standard error, which is the standard deviation of its sampling distribution. Call this σ_b.

[1] Miller, J. J., Cochlin, L., Clarke, K. & Tyler, D. J. Weighted averaging in spectroscopic studies improves statistical power. Magn. Reson. Med. 78, 2082-2094 (2017). doi:10.1002/mrm.26615

Additionally, in order to estimate concentrations of intracellular phosphorus compounds, it is necessary to calculate the approximate intracellular volume of the heart from its mass, m. For this work, following on from others, we have determined it via reference to the specific gravity of myocardial tissue, $s_g = 1.05\,\mathrm{g/ml}$. An intracellular volume fraction of $v_f = 0.48$ additionally is required to correct for extracellular/intracellular fluid in the wet heart weight factors. Depending upon the scanner configuration, a final correction may be required to correct for averaging (as the final PPA spectra may be acquired with fewer averages, which may or may not change the absolute value of the acquired FID); or indeed gain. Ignoring these known constant scalings, the final 'correction' algorithm between derived 31P MRS peak amplitudes x and the concentration c of PPA (in units such as mM) becomes

$$\text{Concentration} = \underbrace{\frac{x}{b/c}}_{\substack{\text{Amplitude-to-volume}\\ \text{correction}}} \times \frac{\overbrace{s_g}^{\substack{\text{Heart intracellular}\\ \text{volume}}}}{m v_f} . \tag{10}$$

This permits the determination of the absolute concentration of metabolites in fully-relaxed spectra.

For the propagation of uncertainty, note that those quantities that are determined on the basis of particularly uncertain things are b and x above. Other quantities are known to sufficient precision to avoid the need to propagate uncertainty quantitatively. This permits the approximate error on the concentration to be simply expressed as

$$\Delta^2_{\text{Concentration}} \approx (\text{Concentration})^2 \left(\left(\frac{\sigma_x}{x} \right)^2 + \left(\frac{\sigma_b}{b} \right)^2 \right) \tag{11}$$

Detailed computations of this quantity for each of the datasets presented in this work are provided later, in Section 2.

1.4 Fluxes

For the computation of fluxes, i.e. the synthetic flux $= k_f[\text{PCr}] + k'_f[\text{Pi}]$ and degradative flux $= (k_r + k'_r)[\text{ATP}]$, we furthermore need to consider the uncertainty in the combined rate constants k. The ideal analysis would again compute the CRLBs on fitted peak uncertainties, perform a form of weighted robust nonlinear regression in the context of fitting kinetic model equations with weights of $1/\text{CRLB}^2$. The estimate of the returned parameter of interest from such a fit, i.e. k_f, k'_f or $(k_r + k'_r)$ is then simply used together with the expression Eq. (2) and derived estimates of uncertainty on concentration measurements. Taken together, this gives the results quoted in the main paper that the uncertainty on derived total forward and reverse fluxes are

$$\begin{aligned}
&\text{Synth. Flux} = k_f[\text{PCr}] + k_f'[\text{Pi}] \\
&\Rightarrow \frac{\sigma_{\text{Synth.}}}{\text{Synth}} = \sqrt{\left(\frac{\sigma_{[\text{Pi}]}}{[\text{Pi}]}\right)^2 + \left(\frac{\sigma_{[\text{PCr}]}}{[\text{PCr}]}\right)^2 + \left(\frac{\sigma_{k_f}}{k_f}\right)^2 + \left(\frac{\sigma_{k_f'}}{k_f'}\right)^2} \qquad (12) \\
&\text{Deg. Flux} = (k_r + k_r')[\text{ATP}] \\
&\Rightarrow \frac{\sigma_{\text{Deg.}}}{\text{Deg.}} = \sqrt{\left(\frac{\sigma_{k_r+k_r'}}{k_r + k_r'}\right)^2 + \left(\frac{\sigma_{[\text{ATP}]}}{[\text{ATP}]}\right)^2}. \qquad (13)
\end{aligned}$$

We note further that while this truncated local-expansion based method is appropriate in the case where errors are small (as they are here), other methods are available and may be less subject to the bias. Although more computationally intensive, full Monte Carlo simulations or other methods (such as importance or interval analysis) may also be appropriate.

2 Individual Results

These uncertainties are now reported in full for each heart considered in the main manuscript. Whilst 31P MRS is inherently a low SNR technique, we highlight again how the main 'point' of the manuscript is the novel RF pulse design method designed for saturation transfer experiments; nevertheless, human experiments with similar pulses will be subject to uncertainty in much the same way.

Measurements and uncertainties computed during the determination of absolute concentration from individual peak amplitudes are shown below, in Tables S2, S4, S6, S8 and S10, with a summary statement on the determined concentrations and uncertainties in Tables S3, S5, S7, S9 and S11. They are initially summarized in Table S1, and it is apparent that the relative error in inorganic phosphate is larger than that of other peaks (a one-way anova reveals a p-value < 0.05). This is arguably unsurprising, reflecting the greater uncertainty in quantifying smaller peaks.

Computing similar fluxes for all spectral time-courses returned in the process of estimating k_f, k_f' and $(k_r + k_r')$, we arrive at the figure quoted in the main manuscript. Detailed spectral information on each time-point is not included for reasons of space.

	Relative error, i.e. $\Delta_{concentration}/Concentration$				
Peak Name	β-ATP	α-ATP	γ-ATP	PCr	P_i
Mean	0.167	0.136	0.103	0.0775	0.276
SD	0.0703	0.0426	0.0455	0.0354	0.184
SEM	0.0315	0.0190	0.0204	0.0158	0.0824

Tab. S1. Summary uncertainty values in derived concentrations.

2.1 Heart 1

Quantity	Value	Unit
Heart Mass	1.916	g
PPA Concentration	100	mM
Specific gravity of tissue	1.05	g/mL
Intracellular volume fraction	0.48	%
Average Correction Factor	4	
PPA Signal Intensities	Value	CRLB
Baseline	8.78	0.18
+100 µl	13.2	0.19
+200 µl	17.0	0.19
Slope	0.0409	a.u. / ul
Regression uncertainty	0.00180	a.u. / ul
PPA Contains	0.0001	mmol / ul
Conversion Factor	409	a.u. / mmol
Heart Intracellular Volume	0.000876	litres

Tab. S2. Summary of parameters (Heart 1)

	Fitted Signals (au)	Conc. (mM)	CRLB (Signals) (au)	Δ Conc (mM)
β-ATP	8.65	6.03	0.650	0.526
α-ATP	10.2	7.14	0.897	0.700
γ-ATP	10.8	7.56	0.542	0.503
PCr	15.0	10.4	0.370	0.526
Pi	9.93	6.93	1.12	0.836
PCr/γ – ATP	1.38	1.38	0.0776	
PCr / ATP	1.51	1.51	0.0732	

Tab. S3. Raw metabolite MRS peak amplitudes and CRLBs, and determined metabolite concentration and ΔConcentration. (Heart 1)

2.2 Heart 2

Quantity	Value	Unit
Heart Mass	1.261	g
PPA Concentration	100	mM
Specific gravity of tissue	1.05	g/mL
Intracellular volume fraction	0.48	%
Average Correction Factor	4	
PPA Signal Intensities	Value	CRLB
Baseline	10.7	0.0558
+100 µl	13.9	0.0634
+200 µl	16.4	0.0656
Slope	0.0286	a.u. / ul
Regression uncertainty	0.00190	a.u. / ul
PPA Contains	0.0001	mmol / ul
Conversion Factor	286	a.u. / mmol
Heart Intracellular Volume	0.000576	litres

Tab. S4. Summary of parameters (Heart 2)

	Fitted Signals (au)	Conc. (mM)	CRLB (Signals) (au)	Δ Conc (mM)
β-ATP	3.43	5.20	0.519	0.858
α-ATP	4.71	7.13	0.663	1.11
γ-ATP	4.64	7.02	0.463	0.841
PCr	7.23	11.0	0.333	0.883
Pi	4.63	7.01	0.812	1.31
$PCr/\gamma - ATP$	1.56	1.56	0.172	
PCr / ATP	1.70	1.70	0.150	

Tab. S5. Raw metabolite MRS peak amplitudes and CRLBs, and determined metabolite concentration and ΔConcentration. (Heart 2)

2.3 Heart 3

Quantity	Value	Unit
Heart Mass	1.155	g
PPA Concentration	100	mM
Specific gravity of tissue	1.05	g/mL
Intracellular volume fraction	0.48	%
Average Correction Factor	4	
PPA Signal Intensities	Value	CRLB
Baseline	9.39	0.188
+100 µl	13.8	0.190
+200 µl	16.7	0.194
Slope	0.0368	a.u. / ul
Regression uncertainty	0.00451	a.u. / ul
PPA Contains	0.0001	mmol / ul
Conversion Factor	367	a.u. / mmol
Heart Intracellular Volume	0.000528	litres

Tab. S6. Summary of parameters (Heart 3)

	Fitted Signals (au)	Conc. (mM)	CRLB (Signals) (au)	Δ Conc (mM)
β-ATP	4.17	5.37	0.583	0.998
α-ATP	6.00	7.72	0.713	1.32
γ-ATP	5.36	6.90	0.530	1.09
PCr	7.88	10.2	0.385	1.34
Pi	3.40	4.38	0.563	0.902
PCr/γ – ATP	1.47	1.47	0.163	
PCr / ATP	1.52	1.52	0.129	

Tab. S7. Raw metabolite MRS peak amplitudes and CRLBs, and determined metabolite concentration and ΔConcentration. (Heart 3)

2.4 Heart 4

Quantity	Value	Unit
Heart Mass	1.10	g
PPA Concentration	100	mM
Specific gravity of tissue	1.05	g/mL
Intracellular volume fraction	0.48	%
Average Correction Factor	4	
PPA Signal Intensities	Value	CRLB
Baseline	9.55	0.242
+100 µl	13.0	0.226
+200 µl	15.860	0.225
Slope	0.0315	a.u. / ul
Regression uncertainty	0.00172	a.u. / ul
PPA Contains	0.0001	mmol / ul
Conversion Factor	315	a.u. / mmol
Heart Intracellular Volume	0.000504	litres

Tab. S8. Summary of parameters (Heart 4)

	Fitted Signals (au)	Conc. (mM)	CRLB (Signals) (au)	Δ Conc (mM)
β-ATP	3.48	5.47	0.629	1.03
α-ATP	4.14	6.51	0.696	1.15
γ-ATP	5.81	9.12	0.545	0.991
PCr	8.01	12.6	0.436	0.970
Pi	3.32	5.21	0.624	1.02
PCr/$\gamma-$ATP	1.79	1.38	0.151	
PCr / ATP	5.81	1.79	0.176	

Tab. S9. Raw metabolite MRS peak amplitudes and CRLBs, and determined metabolite concentration and ΔConcentration. (Heart 4)

2.5 Heart 5

Quantity	Value	Unit
Heart Mass	1.2	g
PPA Concentration	100	mM
Specific gravity of tissue	1.05	g/mL
Intracellular volume fraction	0.48	%
Average Correction Factor	4	
PPA Signal Intensities	Value	CRLB
Baseline	10.1	0.171
+100 µl	14.3	0.171
+200 µl	18.0	0.176
Slope	0.0393	a.u. / ul
Regression uncertainty	0.00158	a.u. / ul
PPA Contains	0.0001	mmol / ul
Conversion Factor	393	a.u. / mmol
Heart Intracellular Volume	0.000549	litres

Tab. S10. Summary of parameters (Heart 5)

	Fitted Signals (au)	Conc. (mM)	CRLB (Signals) (au)	Δ Conc (mM)
β-ATP	1.96	2.27	0.467	0.549
α-ATP	3.66	4.24	0.720	0.852
γ-ATP	5.14	5.96	0.542	0.672
PCr	11.9	13.8	0.330	0.672
Pi	2.57	2.98	0.675	0.791
PCr/γ − ATP	2.31	2.31	0.253	
PCr / ATP	3.31	3.31	0.327	

Tab. S11. Raw metabolite MRS peak amplitudes and CRLBs, and determined metabolite concentration and ΔConcentration. (Heart 5)

List of Tables

S1 Summary uncertainty values in derived concentrations. 5
S2 Summary of parameters (Heart 1) 6
S3 Raw metabolite MRS peak amplitudes and CRLBs, and determined metabolite concentration and ΔConcentration. (Heart 1) . 6
S4 Summary of parameters (Heart 2) 7
S5 Raw metabolite MRS peak amplitudes and CRLBs, and determined metabolite concentration and ΔConcentration. (Heart 2) . 7
S6 Summary of parameters (Heart 3) 8
S7 Raw metabolite MRS peak amplitudes and CRLBs, and determined metabolite concentration and ΔConcentration. (Heart 3) . 8
S8 Summary of parameters (Heart 4) 9
S9 Raw metabolite MRS peak amplitudes and CRLBs, and determined metabolite concentration and ΔConcentration. (Heart 4) . 9
S10 Summary of parameters (Heart 5) 10
S11 Raw metabolite MRS peak amplitudes and CRLBs, and determined metabolite concentration and ΔConcentration. (Heart 5) . 10
